# Ti-Based Biomedical Material Modified with TiO*_x_*/TiN*_x_* Duplex Bioactivity Film via Micro-Arc Oxidation and Nitrogen Ion Implantation

**DOI:** 10.3390/nano7100343

**Published:** 2017-10-23

**Authors:** Peng Zhang, Xiaojian Wang, Zhidan Lin, Huaijun Lin, Zhiguo Zhang, Wei Li, Xianfeng Yang, Jie Cui

**Affiliations:** 1 Institute of Advanced Wear & Corrosion Resistant and Functional Materials, Jinan University, Guangzhou 510632, China; tzhangpeng@jnu.edu.cn (P.Z.); xiaojian.wang@jnu.edu.cn (X.W.); linzd@jnu.edu.cn (Z.L.); hjlin@jnu.edu.cn (H.L.); zhigzhang@jnu.edu.cn (Z.Z.); liweijn@aliyun.com (W.L.); 2 Analytical and Testing Center, South China University of Technology, Guangzhou 510640, China

**Keywords:** titanium, titania, micro-arc oxidation, ion implantation, biotribological properties

## Abstract

Titanium (Ti) and Ti-based alloy are widely used in the biomedical field owing to their excellent mechanical compatibility and biocompatibility. However, the bioinert bioactivity and biotribological properties of titanium limit its clinical application in implants. In order to improve the biocompatibility of titanium, we modified its surface with TiO*_x_*/TiN*_x_* duplex composite films using a new method via micro-arc oxidation (MAO) and nitrogen ion implantation (NII) treatment. The structural characterization results revealed that the modified film was constructed by nanoarrays composed of TiO*_x_*/TiN*_x_* composite nanostitches with a size of 20~40 nm. Meanwhile, comparing this with pure Ti, the friction property, wear resistance, and bioactivity were significantly improved based on biotribological results and in vitro bioactivity tests.

## 1. Introduction

Titanium alloys have been widely used in the skeletal system of the human body as constituents of reconstructive devices (e.g., hip or knee join replacement implant) or fracture fixation products (e.g., bone plates, screws, and nails) [1,2,3,4,5]. The stability of the implant-bone interface is of great importance for a successful bone restoration or bone replacement [6,7,8,9]. The oxide films covering titanium implant surfaces have attracted extensive research interest as they are demonstrated to be crucial for fast osseointegration [10,11,12]. In particular, titanium dioxide (TiO_2_) thin films on Ti alloys could lead to many desirable properties such as excellent biocompatibility, blood compatibility, corrosion resistance, excellent bonding strength with the substrate, and negative surface charge in physiological solution [13].

To obtain a ceramic-like TiO_2_ film on the implant surface, micro-arc oxidation (MAO) is one of the most economic choices; thus it is easy to adopt for mass production. Another advantage of MAO is that it can be applied to an implant with complex structures and leads to a uniform oxide layer. By applying a positive voltage to Ti alloy implants, a TiO_2_ layer could be obtained in an electrolyte [14,15]. This layer is beneficial to cell attachment and bone growth and also shows a better apatite forming ability than nature oxide film on a pure titanium surface [16]. The characteristics of the titanium surface can affect cell proliferation and differentiation; therefore, the choice of the surface modification is crucial to ensure the quality of the process of the formation of new tissues [17,18]. The surfaces of titanium biomaterials with different porosity or structures would offer a superior performance in supporting cell growth than a common tissue culture plate [19].

In current practice, many failures of titanium implants have been found to be related to excessive wear of the implant material [20]. Clinical experience showed that pure Ti and its alloy were known to be more susceptible to wear than stainless steels, which resulted in greater amounts of metallic particles for a loose functional implant [21]. The worn metallic particles might cause local irritations or systemic effects and even the removal of implants [22,23,24]. To improve the wear resistance of the pure titanium surface, titanium nitride (TiN) has been proposed for orthopedic and dental implants due to its high hardness and remarkable resistance to wear and corrosion [25,26]. Moreover, TiN surface has been found to beneficial for the spontaneous nucleation of calcium phosphate [27].

To enhance titanium implants’ mechanical, tribological, and biological properties and to improve their friction and wear properties in the human body and their long-term performance, it is proposed to coat a film with multifunctional properties by combining different surface modification techniques. TiO_2_/TiN duplex films combine the advantages of TiO_2_ and TiN. On one side, a titanium oxide layer on the outer surface could get high blood compatibility. On the other side, the TiN films between the TiO_2_ and the titanium alloy substrate might improve the wear resistance and the adherence strength between the deposited films on the titanium implants.

In this paper, a Ti-based bioactive material with TiO*_x_*/TiN*_x_* duplex bioactivity films was synthesized by a new modification method via micro-arc oxidation and nitrogen ion implantation (MAO-NII). Firstly, porous ceramic-like TiO_2_ films were formed on the titanium substrate by MAO treatment. The TiO_2_ films were then treated by nitrogen ion implantation with different nitrogen ion doses. The structural characteristics of the TiO*_x_*/TiN*_x_* composite modified film (including morphology, phase component, and element composition) were studied. The in vitro bioactivities of the coated specimens were investigated by immersing them in simulated body fluid (SBF) and by examining the apatite formation on their surfaces. Additionally, cell culturing was carried out to study the cyto-compatibility of the duplex films.

## 2. Experimental Procedure

### 2.1. Preparation (MAO and NII Treatments)

Commercially available pure titanium alloys (TA2, purchased from the Northwest Non-ferrous Institute of Technology in Xi’an, China), were used as substrates in the current study. The titanium samples were cut into plates with a size of 15 mm × 10 mm × 2 mm. The surfaces of the plates were abraded with silica papers of 200, 400, 600, 800, and 1200 grit in turn and washed in an ultrasonic bath for 20 min with acetone, ethanol, and de-ionized water, respectively. The titanium plates were then dried in an oven at 40 °C. Micro-arc oxidation (MAO) was carried out using an alternating current-type high power supply (PN-III). The Ti plates served as the anode electrodes, and a stainless steel plate was used as the counter electrode. 0.2 M of calcium acetate monohydrate ((CH_3_COO)_2_Ca·H_2_O, CA) and 0.02 M of β-glycerophosphoric acid disodium salt pentahydrate (C_3_H_7_Na_2_O_6_P·5H_2_O, β-GP) were used as electrolytes. After being treated at 350 V for 5 min, a porous TiO_2_ film formed on the surface of the Ti substrate.

Nitrogen ion implantation (NII) treatment was then performed by using ion implantation equipment with a Kaufman gas ion source (Southwestern Institute of Physics, Chengdu, China). The initial gas pressure in the implantation chamber was under 3 × 10^−3^ Pa. Nitrogen was implanted into the above TiO_2_ films to produce MAO-NII modified samples with an acceleration energy of 80 keV and implantation doses of 0.1, 0.5, 1.0, 5.0, 10, and 20 × 10^17^ ions/cm^2^. The corresponding samples were denoted as Ti-MAO-N0.1 Ti-MAO-N0.5, Ti-MAO-N1.0, Ti-MAO-N5.0, Ti-MAO-N10, and Ti-MAO-N20, respectively.

### 2.2. Structure Characterization

The morphologies of the specimen before and after soaking in SBF were examined by scanning electron microscopy (SEM, ZEISS SUPRA 40, Oberkochen, Germany) and transmission electron microscopy (TEM, JEM-2100F, Tokyo, Japan). For the as prepared sample with kind conductivity, the specimens, after being rinsed and dried, were directly sent for SEM observation. A small amount of powder scraped from the specimen by a diamond knife was dispersed on a micro grid and sent for TEM observation under 200 kV. After SBF and cell tests with poor conductivity, the samples must be rinsed, dried, and sprayed with gold. The phase compositions were characterized by X-ray diffractions (XRD, D/Max 2400 V, Rigaku, Tokyo, Japan) using Cu K_α_ radiation in the regular range of 2*θ* = 20°~80°, with an accelerating voltage of 36 kV and a current of 100 mA.

### 2.3. Friction and Wear Test Bioactivity Evaluation

A wet friction and wear test was carried on a ball-on-disc high speed reciprocation friction and wear tester (MFT-R4000, Lan zhou, China, Lanzhou Institute of Chemical Physics, Chinese Academy of Sciences). An Al_2_O_3_ ceramic ball with a diameter of 4.0 mm and a radius of 0.032 μm was used as the friction match pair. 50% calf serum (the ratio of calf serum with demonized water is 1:1) was chosen as the lubricant. During the friction tests, the applied normal load was 200 g, with a reciprocation frequency of 2 Hz. The reciprocation distance was fixed as 5 mm, and the friction time was 1 h. Each material is tested for five parallel samples, and the average value is taken by removing the highest and lowest values.

The SBF solution was prepared on the basis of Kokubo’s recipe [22] for the bioactivity evaluation. The SBF tests were referenced according to Hiroaki’s method [28]. The volume of SBF that is used for testing was determined by Equation (1).

*Vs* = *Sa*/10 (1)

where *Vs* is the volume of SBF (mL) and *Sa* is the apparent surface area of the specimen (mm^2^) [28]. Put the calculated volume of SBF into a plastic bottle or beaker. After heating the SBF to 36.5 °C, the specimens were submerged pensile in the SBF to avoid over-saturation. After soaking for 24 days, the specimens were washed gently with distilled water and dried at room temperature.

The MC3T3 E1 cell line from embryonic osteoblasts of mouse embryos was used for cytotoxicity tests. The culture medium consisted of alpha-minimum essential medium (α-MEM) supplemented with 10% fetal bovine serum (FBS), 100 U·mL^−1^ of penicillin, and 100 μg·mL^−1^ of streptomycin sulfate. The experiments were conducted in an incubator at 37 °C, with a humidified atmosphere of 95% air and 5% CO_2_ for two days. The specimens were sterilized by heating at 180 °C for 1 h. The cells were fixed with 5 mL of 10% formalin for 30 min, stained with 8 mL of 0.15% methylene blue for an additional 30 min, washed thoroughly with different concentrations of alcohol, and dried [29,30].

## 3. Results and Discussion

**Figure 1:**
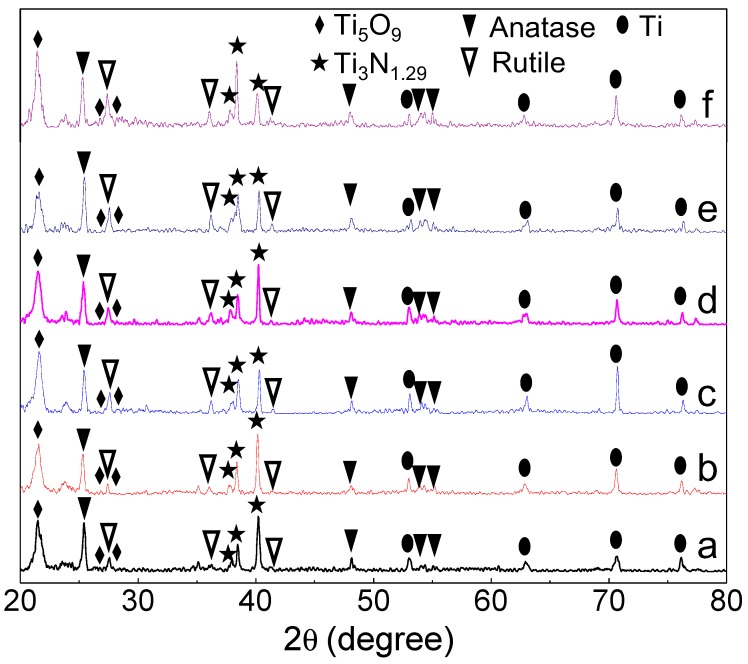
(**a**–**f**) X-ray diffraction (XRD) patterns of the micro-arc oxidation (MAO) specimens with different N implantation doses: Ti-MAO-N0.1, Ti-MAO-N0.5, Ti-MAO-N1.0, Ti-MAO-N5.0, Ti-MAO-N10, and Ti-MAO-N20.

The XRD patterns of all the specimens treated with the MAO-NII procedure are shown in Figure 1. In addition to the anatase and rutile TiO_2_ obtained in the MAO process, unsaturated titanium oxide and titanium nitride were also detected. The XRD peaks marked with rhombi in all patterns, as shown in Figure 1, could be indexed to an unsaturated titania of Ti_5_O_9_. The XRD peaks marked with stars corresponded to Ti_3_N_1.29_. It can be concluded that titanium nitrides and various titanium oxides coexist in the modified layer after nitrogen ion implantation. Namely, a TiO*_x_*/TiN*_x_* composite film on the surface of Ti substrate has been obtained.

**Figure 2:**
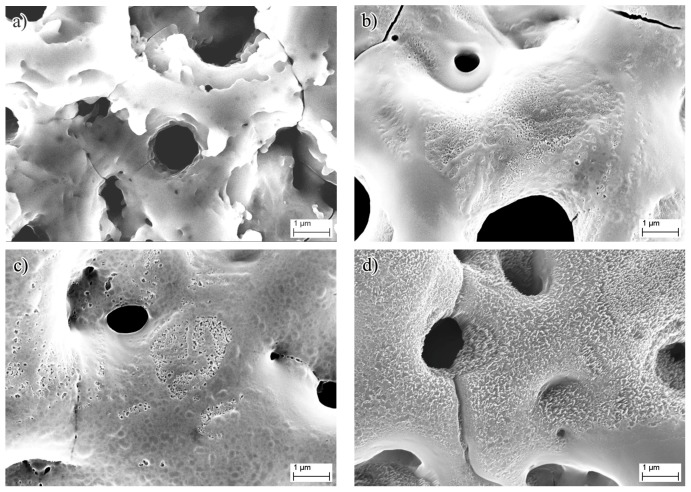

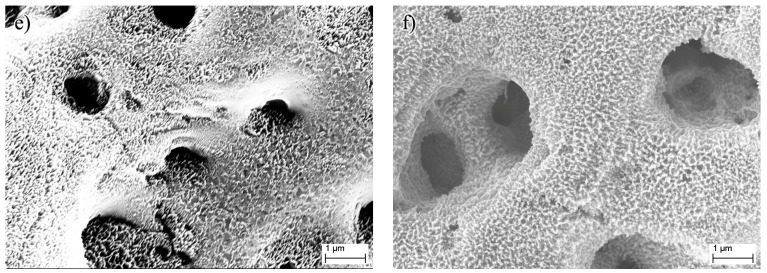
Surface morphologies of the micro-arc oxidation and nitrogen ion implantation (MAO-NII) modified specimens with different N ion implantation doses: (**a**) Ti-MAO-N0.1; (**b**) Ti-MAO-N0.5; (**c**) Ti-MAO-N1.0; (**d**) Ti-MAO-N5.0; (**e**) Ti-MAO-N10; and (**f**) Ti-MAO-N20.

Then the morphology of the composite films was investigated by SEM. The top-view surface morphology of the samples implanted with different nitrogen doses is shown in Figure 2. The typical characteristics of MAO-NII modified films with micron-sized pores are shown in Figure 2a. The wall surfaces of the holes are very slick when the N implantation dose is very low (0.1 × 10^17^ ions/cm^2^). By increasing the N implantation dose, the surface morphology was changed in varying degrees. Some small pores appeared on the surface of the MAO modified layer, as shown in Figure 2b,c. When the N implantation dose was 5.0 × 10^17^ ions/cm^2^, most of the surface was not slick anymore, as shown in Figure 2d. This rough morphological feature of the surface is made more obvious in Ti-MAO-N10 and Ti-MAO-N20 by continuously increasing the N implantation dose to 10 and 20 × 10^17^ ions/cm^2^ (Figure 2e,f). Thus we found that the surface would become rougher with increasing N implantation doses.

**Figure 3:**
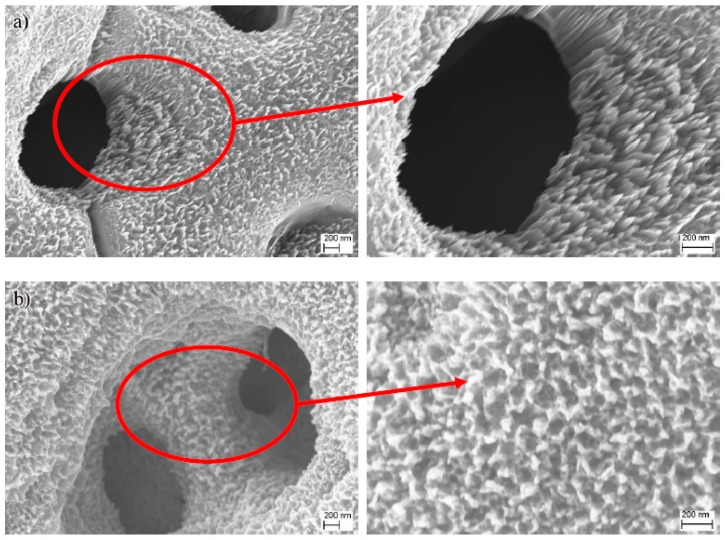
Surface images of nitrogen ion implantation MAO specimen under high magnification morphology: (**a**) Ti-MAO-N10 and (**b**) Ti-MAO-N20.

Interestingly, unique nanoarrays composed of vertically aligned nanostitches with a size of 20 to 40 nm were found in Ti-MAO-N10 by a high magnification morphological observation under SEM, as shown in Figure 3a. Moreover, the sharp tips of the nanostitches were only about 5~10 nm. However, the nanostitch array structure disappeared when the N implantation dose was increased to 20 × 10^17^ ions/cm^2^, as shown in Figure 3b, and it was replaced by many small pores with a size of 30~60 nm. Presumably, the nanostitch array structure collapsed due to the high ion implant energy from the increase of the implantation dose.

**Figure 4:**
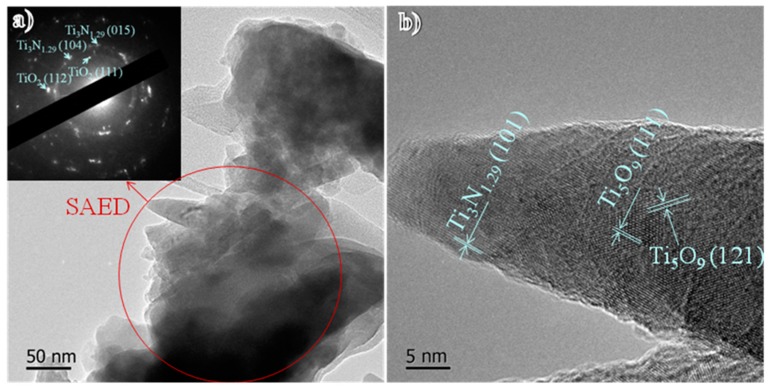
(**a**) Bright field transmission electron microscopy (TEM) image with selected area of the electron diffraction (SAED) pattern inset and (**b**) high resolution TEM (HRTEM) image of TiO*_x_*/TiN*_x_* film of Ti-MAO-N1.0.

**Figure 5:**
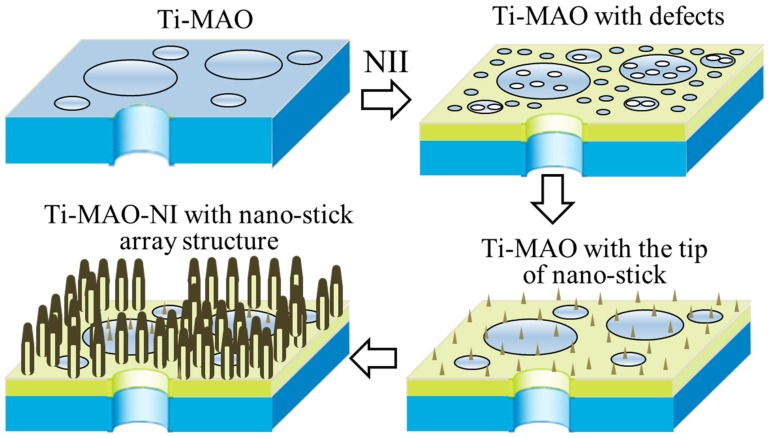
Schematic diagram of the growth mechanism of the TiO*_x_*/TiN*_x_* composite modification layer with a nanostitch array structure during NII.

To further study the crystallographic structure of the TiO*_x_*/TiN*_x_* composite film, a small amount of powder was scraped from the surface of Ti-MAO-N1.0 for TEM observation. The result is shown in Figure 4. It is proved again the nanostitch structure has a tip diameter of 5 nm and a bottom diameter of about 30 nm. As shown in Figure 4a, the main diffraction spots in the selected area of the electron diffraction (SAED) pattern from a circle area of the film correspond to anatase TiO_2_ and Ti_3_N_1.29_ phases. High resolution TEM (HRTEM) suggests the presence of Ti_3_N_1.29_ and Ti_5_O_9_ respectively within the top and bottom of the nanostitch. Hence, the formation mechanism of the composite modified film with the nanostitch array structure can be speculated based on the above results. As shown in Figure 5, lots of defects such as nanopores appeared with the bombardment of implanted ionic fluxes towards the surfaces at first. Then nanostricks formed and grew to form nanoarrays, which should be mainly composed of unsaturated titania due to the O atoms in TiO_2_ being be partly removed by implanted N ions during the NII process. Meanwhile, some of the O atoms of titanium oxide on the surface nanostitches, especially on the top surface, were replaced by N, resulting in the nucleation of nano-sized titanium nitride on the tips. However, if the NII dose is too high, the nanostitch array structure will collapse due to the high implantation energy and superheat.

**Figure 6:**
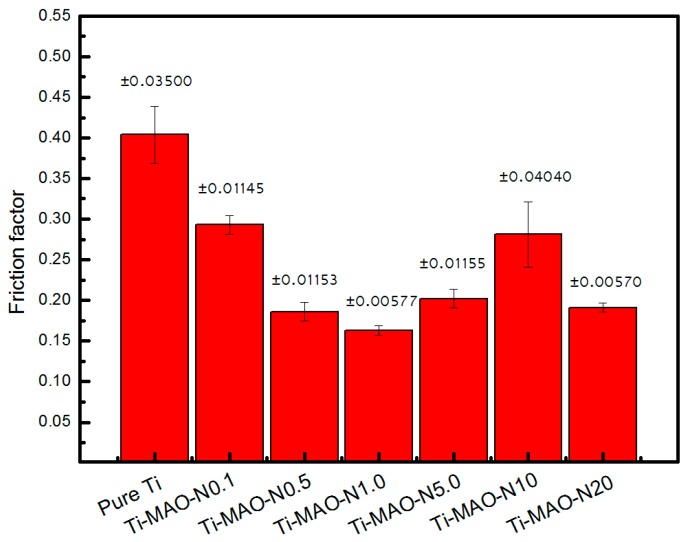
Friction coefficient of MAO specimens with different N doses under 50% mavericks serum lubrication conditions.

The friction factors for specimens after MAO and NII treatment are obtained under 50% mavericks serum lubrication conditions, as shown in Figure 6. It can be found that the friction factors for the treated specimens were all lower than that of the pure titanium (0.404). In addition, the results showed that Ti-MAO-N1.0 (10^17^ ions/cm^2^) exerted the lowest average friction factor, which may lead to the best biological friction performance. As discussed above, the composite modified layer is composed of antatase and rutile TiO_2_, Ti_5_O_9_ and Ti_3_N_1.29_. Ceramic TiO_2_ and titanium nitride are hard phases, which show much higher hardness than pure Ti. As well, the surface hardness increased with the amount of hard nitrides and oxides, which resulted in the reduction of the friction factor. However, the nitrogen ion implantation exists as a saturation injection dose. The increasing amount of implantation will not enhance the surface hardness unlimitedly. In this study, 10^17^ ions/cm^2^ was the best implantation dose, when considering the biological friction performance.

**Figure 7:**
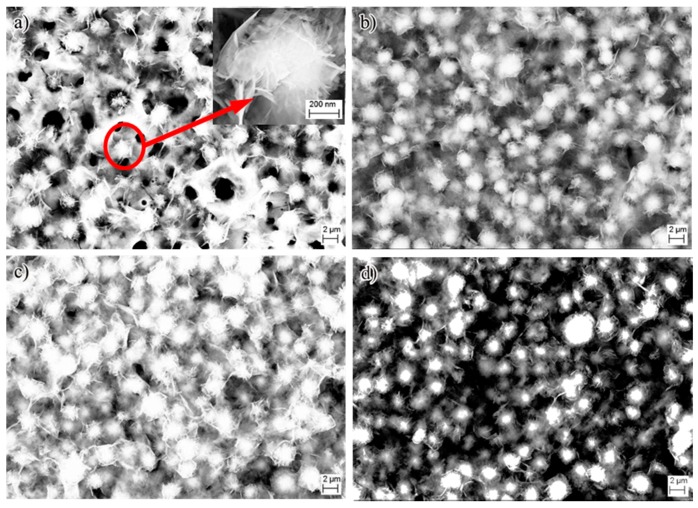
Surface morphology of the samples after soaking in biomimetic mineralization solution for 24 days: (**a**) Ti-MAO-N0.1; (**b**) Ti-MAO-N1.0; (**c**) Ti-MAO-N5.0; and (**d**) Ti-MAO-N20.

In addition to biological friction, biological activity is also concerned in this study. The specimens treated by MAO and NII were immersed in biomimetic mineralization solution for 24 days. The samples after soaking were examined by SEM, and the results are shown in Figure 7. SEM images showed that all the sample surfaces were almost fully covered with ball–shaped particles. There are some nano-flakes on the surfaces of the globular objects, which is similar to the urchin shown with the red arrow. Compared with the SBF soaking result of pure Ti, shown in Figure S1a in the supporting information, the bioactivity of the Ti-MAO-NI (Figure S1b,c) samples is improved. Additionally, combed with the energy dispersive spectrometer (EDS) results shown in Table 1, it can be predicted that Ca and P are fully deposited on the surfaces of the samples after MAO and NII. It can be predicted that apatite was deposited, which is one of the important human bone inorganic compositions [30,31]. Hence, this illustrates a good ability to inducer phosphate deposition, which also leads to good in vitro bioactivity and also indicates that all the samples after MAO and NII modification show good bioactivity. Furthermore, it can also found that the change of the content of Ca and P is consistent with the content of N implantation. Hence, it can be suspected that titanium nitride is beneficial to apatite deposition and facilitates bioactivity.

**Figure 8:**
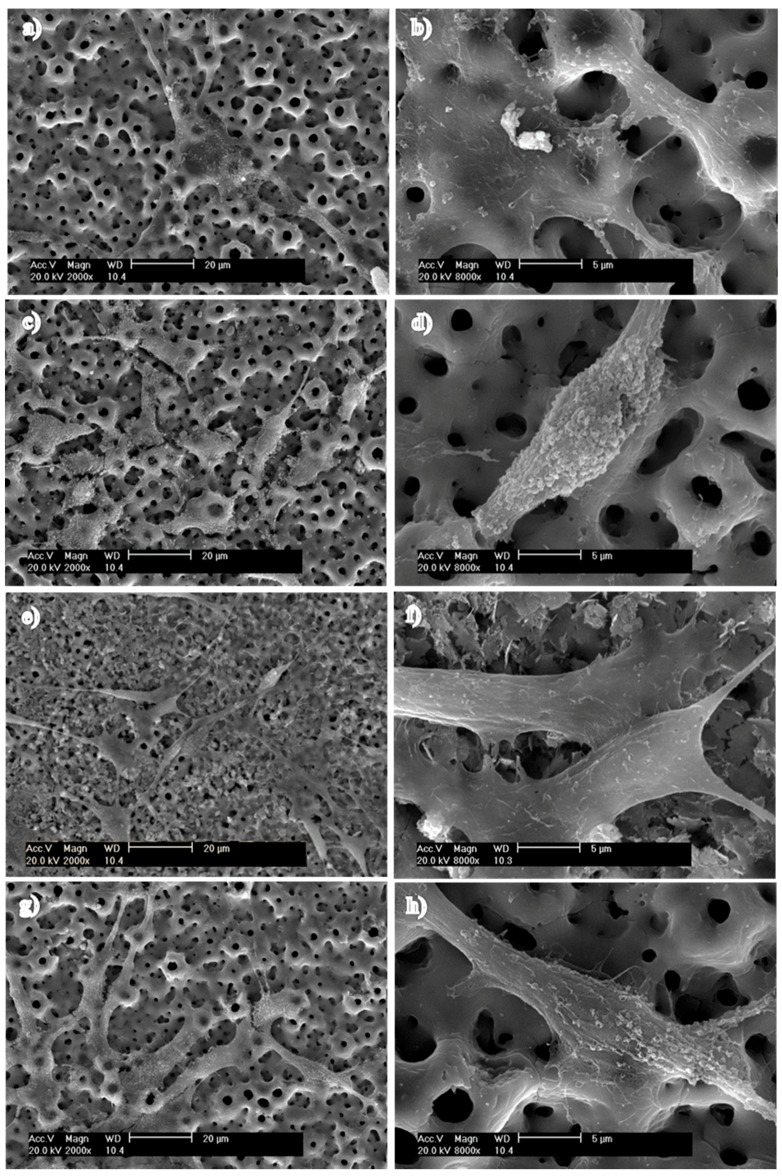
The surface morphology of MAO specimens with different N implantation doses after cell culturing for two days: (**a**,**b**) Ti-MAO-N0.1; (**c**,**d**) Ti-MAO-N1.0; (**e**,**f**) Ti-MAO-N5.0; (**g**,**h**) Ti-MAO-N20.

The cyto-compatibility of the samples was also evaluated by cell cultures on the different surfaces. The surface morphology of the Ti-MAO-NI specimens after cell culturing for two days is shown in Figure 8 and Figure S2 in the supporting information. The alkaline phosphatase activity of osteoblasts on pure Ti and Ti-MAO-N1.0 surface was shown in Figure S3. The cells spread well and showed a good attachment, with a plumpness and a polygon shape on the specimen surface. The cells also developed numerous filopodia, sensing the different surface specimens. Compared with the Ti-MAO-N0.1, Ti-MAO-N0.5, Ti-MAO-N1.0, Ti-MAO-N10, and Ti-MAO-N20, the amount of cells in Ti-MAO-N5.0 is the most. Thus, it can be concluded that cyto-compatibility can be promoted by NII treatment.

The porosity and nanostitch array structures of the samples can affect cell proliferation and differentiation, which is crucial to ensuring the quality of the process of the formation of new tissues. The morphology of these surfaces could have a mechanical influence on the cells used. It should be further investigated [32].

## 4. Conclusions

A composite modified film has been successfully synthesized on the surface of Ti alloy by MAO-NII treatment. The as-prepared film is composed of titania (anatase and rutile TiO_2_), unsaturated titania (Ti_5_O_9_), and titanium nitride (Ti_3_N_1.29_). The surface exhibits a unique nanostitch array structural feature, the growth mechanism of which has been studied by detailed TEM characterization. The friction factor of the composite modified film was much lower than that of pure titanium (0.404). Bioactivity and cellular compatibility have improved greatly with the MAO and NII treatment compared with pure Ti. Additionally, it was also found that the nanostitch array structure collapsed due to the high implantation energy and superheat when the implantation dose rose to 20 × 10^17^ ions/cm^2^.

## Figures and Tables

**Table 1:** Energy dispersive spectrum (EDS) analysis results of samples with different doses of N ion implantation after soaking in biomimetic mineralization solution for 24 days.

Sample Name	Element Content (wt %)				
	Ti	O	Ca	P	N
Ti-MAO-N0.1	41.12	43.39	10.30	3.47	1.72
Ti-MAO-N1.0	42.30	41.19	11.01	3.48	2.02
Ti-MAO-N5.0	36.79	40.52	14.43	4.94	3.32
Ti-MAO-N20	51.89	36.05	5.87	4.14	2.05

## Supplementary Materials

The following are available online at http://www.mdpi.com/2079-4991/7/10/343/s1, Figure S1: Surface morphology of pure Ti (a), Ti-MAO-N0.5(b) and Ti-MAO-N10 (c) after soaking in biomimetic mineralization solution for 24 days, Figure S2: The surface morphology of MAO specimens with different N implantation dose after cell cultured for 2 days: Ti-MAO-N0.5 (a,b), Ti-MAO-N10 (c,d), Figure S3: The alkaline phosphatase activity of osteoblasts on pure Ti and Ti-MAO-N1.0 surface.
