# Supplementary material for: Ti-Based Biomedical Material Modified with TiOx/TiNx Duplex Bioactivity Film via Micro-Arc Oxidation and Nitrogen Ion Implantation
Source: Nanomaterials (Basel). 2017 Oct 23;7(10):343. doi: 10.3390/nano7100343 (PMC5666508; doi:10.3390/nano7100343)
Supplement: Supplementary file 1 [file nanomaterials-07-00343-s001.pdf]

Supporting information

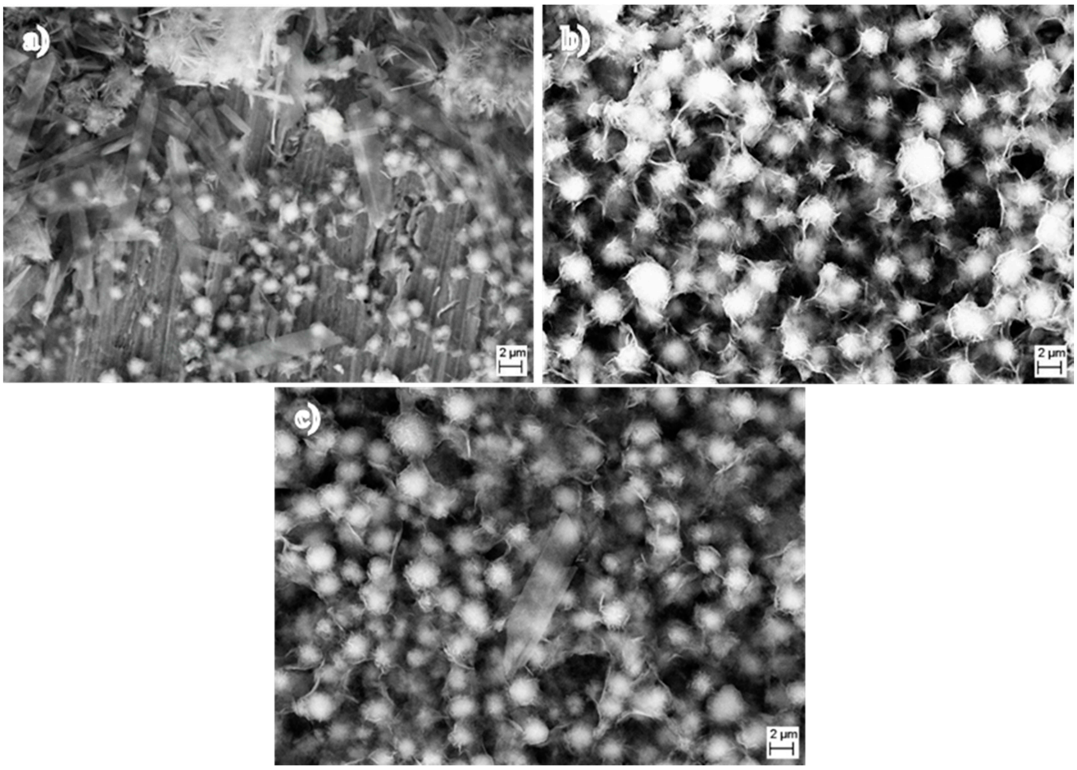

Fig. S1 Surface morphology of pure Ti (a), Ti-MAO-N0.5(b) and Ti-MAO-N10 (c) after soaking in biomimetic mineralization solution for 24 hour.

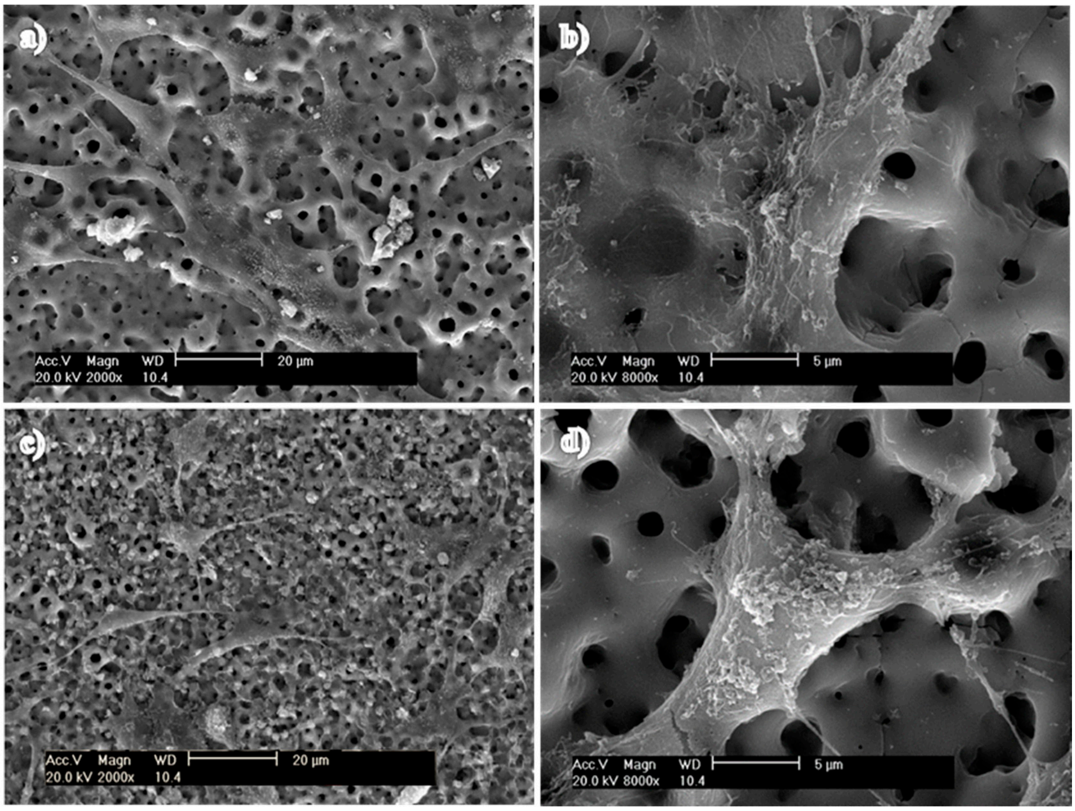

Fig. S2. The surface morphology of MAO specimens with different N implantation dose after cell cultured for 2 days: Ti-MAO-N0.5 (a,b), Ti-MAO-N10.0 (c,d)

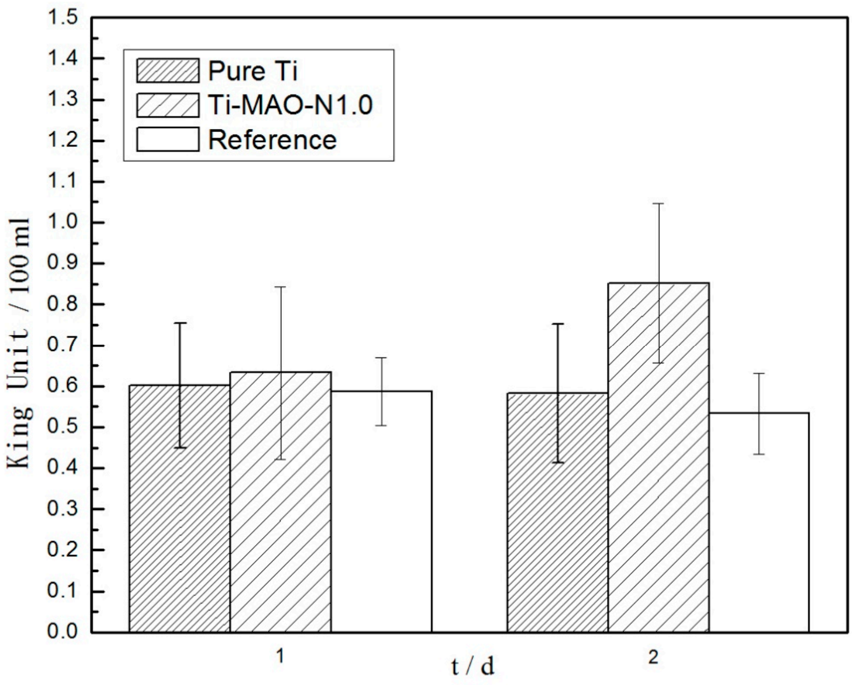

Fig. S3. The alkaline phosphatase activity of osteoblasts on pure Ti and Ti-MAO-N1.0 surface

The ALP activity reflects the level of differentiation in osteoblasts. Measuring ALP activity in osteoblasts culture medium is a simple, fast and accurate assay method. We have done ALP activity assays to determine the cytotoxicity of the materials. Result manifested that most of the test compounds did not have significant effects on ALP activity of osteoblasts at the concentrations about 0.6 king unit per 100ml in primary osteoblasts after 24h treated. While after incubating 48h, NII sample showed various ALP activity. Among them Ti-MAO-N1.0 exhibited higher ALP activity than pure titanium and reference. The optimal concentration of Ti-MAO-N1.0 ALP activity is 0.851 king unit per 100ml as shown in Fig. S3.
